# Intra-Minute Cloud Passing Forecasting Based on a Low Cost IoT Sensor—A Solution for Smoothing the Output Power of PV Power Plants

**DOI:** 10.3390/s17051116

**Published:** 2017-05-13

**Authors:** Primož Sukič, Gorazd Štumberger

**Affiliations:** Faculty of Electrical Engineering and Computer Science, University of Maribor, Smetanova 17, SI-2000 Maribor, Slovenia; primoz.sukic2@um.si

**Keywords:** photovoltaic power plant, cloud passing forecasting, algorithm, sensor, Raspberry Pi, camera, wide-angle lens, optical filters, Internet of Things

## Abstract

Clouds moving at a high speed in front of the Sun can cause step changes in the output power of photovoltaic (PV) power plants, which can lead to voltage fluctuations and stability problems in the connected electricity networks. These effects can be reduced effectively by proper short-term cloud passing forecasting and suitable PV power plant output power control. This paper proposes a low-cost Internet of Things (IoT)-based solution for intra-minute cloud passing forecasting. The hardware consists of a Raspberry PI Model B 3 with a WiFi connection and an OmniVision OV5647 sensor with a mounted wide-angle lens, a circular polarizing (CPL) filter and a natural density (ND) filter. The completely new algorithm for cloud passing forecasting uses the green and blue colors in the photo to determine the position of the Sun, to recognize the clouds, and to predict their movement. The image processing is performed in several stages, considering selectively only a small part of the photo relevant to the movement of the clouds in the vicinity of the Sun in the next minute. The proposed algorithm is compact, fast and suitable for implementation on low cost processors with low computation power. The speed of the cloud parts closest to the Sun is used to predict when the clouds will cover the Sun. WiFi communication is used to transmit this data to the PV power plant control system in order to decrease the output power slowly and smoothly.

## 1. Introduction

In recent years, the number of photovoltaic (PV) power plants connected to electricity networks has increased substantially. Clouds moving at high speed in front of the Sun can cause sudden changes in the output power of PV power plants, which can lead to voltage fluctuations and, consequently, to stability problems in the connected electricity networks [1,2,3]. Electricity network reinforcement and the introduction of energy storage systems are two effective, but very costly, measures that can be applied to control the aforementioned problems [3,4,5,6,7]. Similar results can be achieved by a programmed smooth reduction of the PV power plant output power based on the forecasting of cloud movement in the vicinity of the Sun (cloud passing forecasting). This approach is much cheaper than the two previously mentioned ones, but requires a forecast of clouds passing the Sun in the time range of one minute. The forecasting is based on a sequence of photos of the sky, taken at equidistant time intervals of several seconds. The clouds in the photos have to be identified, and then the movement of the clouds is predicted based on the changes of their position in the sky photo sequence. Some of the approaches that can be applied are described in [8,9,10,11,12]. 

The most common sources of photos, images and other data applied to predict the movement of clouds in front of the Sun are satellite cameras, geographically distributed sensor networks and stationary cameras. Satellite images are normally applied to predict the movement of clouds over a broader area in a time interval ranging from 0.5 to 6 h [13,14,15]. They are less suitable for predicting the movement of small and fast moving clouds [16]. Sufficiently dense geographically distributed sensor networks, mostly consisting of pyranometers or PV power plants with a permanent measurement of output power, can normally provide the input data for a short-term prediction of cloud movement with an acceptable accuracy [16,17]. The accuracy of a short-term prediction of cloud movement can be improved further, if it is based on sequences of photos made by a stationary camera. The stationary camera can be fixed, or it can move on a tracking system [18,19,20]. Based on the substantial effort invested in their development, Total Sky Imager [21,22] and UC San Diego Imager [8,23], with wide-angle lenses, are among the most refined and most used cameras in this field of applications. In order to prevent saturation of the CCD sensor in the camera, some solutions are equipped with a shadow band mounted in front of the lens. Its moving part prevents the sunlight from having direct access to the CCD sensor. In this way, the quality of the photo around the Sun is improved, which is essential for the proper recognition of clouds. Unfortunately, the shadow band covers a part of the photo close to the Sun, which, in the case of slow moving clouds, decreases the accuracy of the cloud movement prediction, making this approach less suitable for a prediction of cloud movement in the range of several minutes [8,11,22]. In this time range, the systems without shadow bands or similar optical obstacles, such as San Diego Sky Imager [24], perform much better. The problems caused by the saturation of the CCD sensor and flare can be reduced effectively by a proper use of natural density (ND) and circular polarizing (CPL) filters [25], whilst the use of CCD sensors with 12-bit resolution or better, improves the performance of digital filters applied in image processing [8].

A prediction of cloud movement, based on a sequence of taken photos, normally involves several image processing steps. The recognition of the clouds is normally based on different values of the Red Blue Ratio (RBR) [8,23,26]. In order to predict the cloud movement, the entire photo is discretized into smaller, often square-shaped regions. The areas recognized as clouds inside these square-shaped regions in a sequence of taken photos, are used to determine the cloud motion vectors [8,9,11,27]. These vectors are then used to predict the cloud movement towards the Sun. This prediction can also be called a cloud passing forecasting. In this process, machine learning-based methods have proven to be very effective and accurate [28,29,30]. The random walk mobility model used in mobile telecommunication networks [31,32], where the mobility direction and velocity of each node can be chosen randomly and independently, is not directly applicable in this case.

The existing professional systems for solar irradiance forecasting can provide accurate cloud passing forecasts, but only at high cost. Their price can easily exceed the price of a small PV power plant, suitable for a yearly net self-sufficient electricity supply of a household [24]. Since the applied image processing methods involve the entire photo, the required computational effort is rather high and requires suitable hardware. 

This paper proposes a low-cost system for cloud passing forecasting in the time interval of under one minute. Its only goal is forecasting clouds that will cover the Sun in the next minute. The cloud passing forecasting system generates a trigger signal. It is used in the PV power plant control system and in the inverters as a command to reduce the output power of the PV power plant smoothly, without applying a battery system. The cost of the proposed system is lower than the cost of a single 270 Wp PV module. This is achieved by the use of widely available and cheap hardware components, such as processor units, CCDs with wide-angle lenses and optical filters, and novelties in the field of image processing. Two main novelties are proposed in this paper. The first one is the use of the green and the blue colors for cloud recognition and reduction of flare problems. The second one is image processing, limited to a narrow area around the Sun, which is sufficient for intra-minute cloud passing forecasting and can be performed with a low computational effort.

This paper consists of four sections. The main goals of the paper, references to previous work and the novelties introduced in the paper are described in the Introduction. The section entitled Cloud Passing Forecasting System contains the descriptions of the applied hardware, the algorithms for Sun and cloud recognition, and the algorithm for the prediction of cloud movement in the vicinity of the Sun. The performances of the proposed cloud passing forecasting are demonstrated in the third section, Results, whilst the final conclusions are drawn in the last section. 

## 2. Cloud Passing Forecasting System

The proposed solution for cloud passing forecasting consists of sky image acquisition hardware, described in Section 2.1, and software, described in Section 2.2, Section 2.3 and Section 2.4. The cloud passing forecasting system planning is based on the following criteria:

- It must be able to take photos of the sky independently without sunshades;
- It must also be able to recognize clouds near the Sun, when the Sun is located perpendicularly to a camera;
- No moving parts;
- All hardware and optics must be low-cost;
- The cloud passing forecasting algorithm must be applicable on low-cost IoT modules.

### 2.1. Image Acquisition Hardware

**Figure 1:**
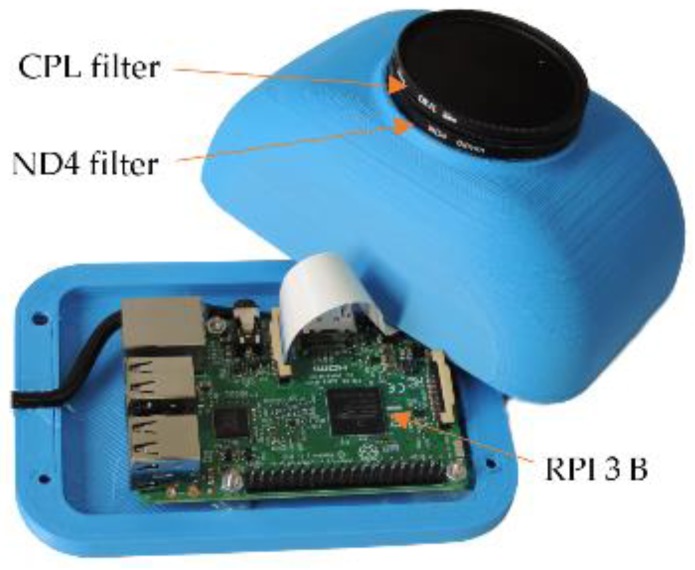
Used hardware.

Based on the given criteria, we have chosen to build up our cloud passing forecasting system around the Raspberry Pi Model B 3 board (Raspberry Pi Foundation, Cambridge, United Kingdom) with a 1.2 GHz 64-bit quad-core ARMv8 CPU and 802.12n Wireless LAN, and the Waveshare RPI Camera (G) (Waveshare, Shenzhen, China), which is based on the OV5647 sensor (OmniVision, Santa Clara, CA, USA). The camera contains a wide-angle lens. When properly oriented, it captures images of the sky with the sun and the clouds around it over the entire part of the day, where reduction of the PV power plant output power could be required. Two optical filters, a circular polarizing filter (CPL) and a natural density (ND 4) filter have been mounted in front of the wide-angle lens. The ND 4 filter reduces the density of the light that falls on the sensor and prevents its saturation and overheating. The CPL filter reduces the glare and reduces the density of the light further [25]. The hardware part of the cloud passing forecasting system is shown in Figure 1, whilst the costs of individual hardware components are shown in Table 1. 

The hardware shown in Figure 1 is used to take sequences of photos. The algorithms, applied on these photos in order to achieve a proper cloud passing forecasting, are described in the next subsections.

### 2.2. Sun Recognition

In order to predict the movement of clouds in the vicinity of the Sun, the location of the sun must be determined in the photos taken. This can be done in two ways. If the system is already calibrated, the position of the Sun in a photo can be determined considering the current time and the Sun’s analytically described trajectory. In the case of an uncalibrated system, or when the offset between the calculated location and the actual location of the Sun in the photo has to be eliminated, the recognition of the Sun in the photo is performed, followed by the determination of its location. For each pixel with the coordinates (x,y) in the photo it is checked if the condition (1) is fulfilled. The pixels that represent the sun have the feature that the value of each RGB (Red Green Blue) color is in the range between 250 and 255. Due to the reflection problems described later in Section 2.3, the red color is not used in the Sun recognition. Thus, each pixel, where the sum of the RGB values for the green and the blue color exceeds 500 (1), can be considered part of the sun, which has been confirmed experimentally:

(1) green(x,y)+blue(x,y) >500

The pixels that fulfill Equation (1) are considered as part of the Sun. Their coordinates (*x*, y) are stored as ($x_{p\_sun}$,$y_{p\_sun}$). The center of gravity of the surface that contains all of the pixels recognized as the Sun is calculated by Equations (2) and (3):

(2) $$x_{ave\_sun}=\frac{\sum_{x_{start}}^{x_{end}}x_{p\_sun}}{n_{p\_sun}}$$

(3) $$y_{ave_{sun}}=\frac{\sum_{y_{start}}^{y_{end}}y_{p_{sun}}}{n_{p_{sun}}}$$

where $n_{p\_sun}$ is the number of pixels recognized as the Sun, whilst $x_{ave\_sun}$ and $y_{ave\_sun}$ are the coordinates of the Sun’s center. The symbols $x_{start}$, $x_{end}$ and $y_{start}$, $y_{end}$ denote the upper and lower bounds for x and y.

**Figure 2:**
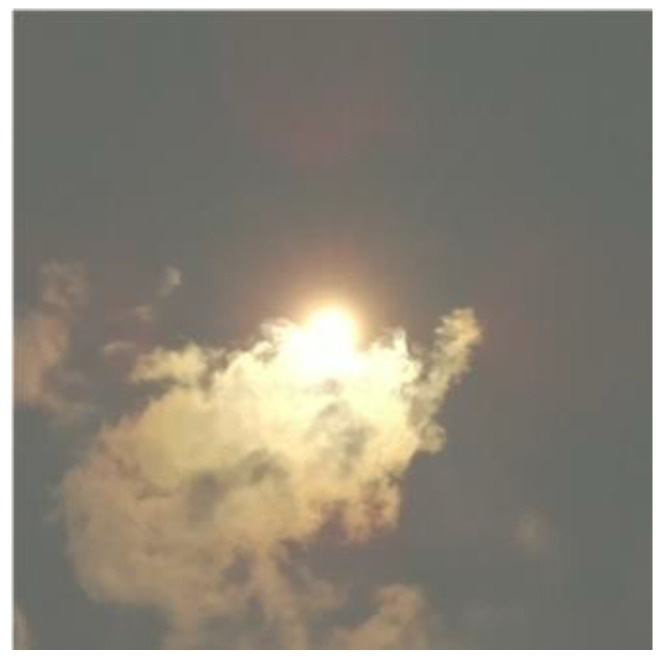
Partially translucent cloud covering the sun.

The sky is often unclear and the Sun can be partially covered by translucent clouds, as shown in Figure 2. This could cause some parts of the clouds covering the Sun being recognized as the Sun, which could shift the center of the area recognized as the Sun from the real center of the Sun in the photo. In order to identify this problem, the maximal and minimal number of pixels in the photo that represents the Sun not covered by translucent clouds is determined empirically, considering the variation of the Sun’s size in a series of photos of the clear sky. 

The number of pixels recognized as the Sun in a photo outside the prescribed limits, means that the Sun is at least partially covered by clouds. In such cases, the Sun’s center coordinates are calculated using the Sun’s trajectory equations [33]. Regardless of how they are determined, the coordinates of the Sun’s center are marked with $\left(x_{sun},y_{sun}\right)$ in the rest of this paper.

The cloud passing forecasting system proposed in this work is limited by the prediction of cloud movement in the vicinity of the Sun in the next minute. Thus, only a narrow area of the photo, which is near the Sun, is considered an area of interest. This area is chosen according to the known coordinates of the Sun’s center in the photo (heliocentric system). In the following text, the term photo refers only to the area of interest, which is actually a cropped down part of the original photo. 

The proposed approach reduces substantially the computation power required for the image processing, enabling the realization of cloud passing forecasting on small and low-cost hardware such as the Raspberry PI Model B 3.

### 2.3. Recognition of Clouds 

**Figure 3:**
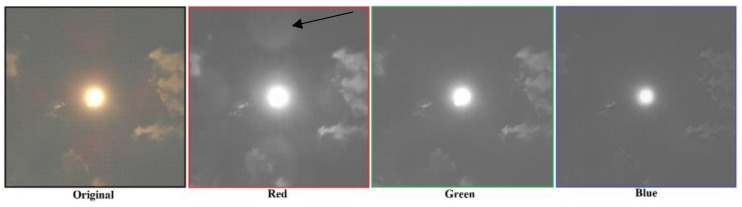
Photo and its decomposition into individual RGB colors.

An appropriate digital filter is applied to recognize the clouds in a photo. Optical lenses pose an additional problem since, under certain conditions, a light reflection appears, which can influence the recognition of the clouds in the photo. The light reflection, which appears mostly on the lens and the optical filters in the red color, is a property of the applied optical system, as shown in Figure 3, where the photo is decomposed into individual RGB colors. The arrow shows the reflection in the red color part of the photo. The reflection is, in this work, treated phenomenologically. This means that the effects of the reflection that appears in the photos taken by the applied optical system, and can disturb the recognition of the Sun and clouds, are eliminated by not using the red color part of the photo in the procedures applied to recognize the Sun and the clouds in the photo.

When reflections are present, the widespread digital filter RBR (Red Blue Ratio) [8,12,23,34,35] does not work well. In the given case, shown in Figure 3, the recognition of clouds, based on the RBR ratio is disturbed due to the reflection present mostly in the red color. The reflection is much less present in the green and the blue colors, where it is almost invisible. Therefore, the recognition of the clouds, based on the ratio between the green and the blue colors, is proposed in the form of a Green Blue Ratio (GBR) digital filter. For each pixel in the photo the values of the green (G) and the blue (B) colors are defined in the range between 0 and 255. Their ratio is compared with the experimentally determined threshold value $GBR_{lim}=1.2$ in (4). The logical values obtained by Equation (4) are inserted in the cloud recognition matrix $C_{r}$. Its elements correspond with individual pixels, where the logical value true = 1 means that the pixel belongs to a cloud:

(4) $$\frac{G}{B}>GBR_{lim}$$

**Figure 4:**
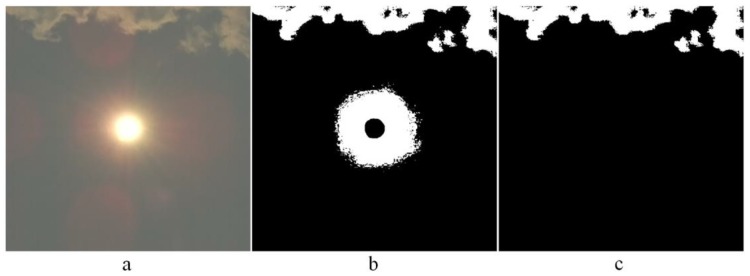
Cloud recognition: (**a**) Photo of the sky, (**b**) Cloud recognition matrix with the area around the sun, (**c**) Area recognized as a cloud.

The fact that the illumination and brightness on the cloud’s edge is increased coincides with the increased GBR value, which makes the recognition of the clouds even easier and more reliable. It also enables the use of very short exposure times (high shutter speeds) in the range of 20–100 µs. Light passing through the atmosphere causes scattering very close to the Sun, which is caused by moisture, temperature, dust particles, etc. Since these effects could influence the proposed cloud recognition, the logical values in the cloud recognition matrix $C_{r}$ in the radius around the Sun’s center that exceeds the Sun’s radius 2 to 4 times, are set to false = 0. All the other values in the cloud recognition matrix remain unchanged. Figure 4a shows a photo of the sky. The cloud recognition matrix, where the clouds and the area around the sun are marked, is shown in Figure 4b. The cloud recognition matrix, where only the areas recognized as clouds are marked, is shown Figure 4c. 

The area recognized as clouds could contain different disturbances caused by birds, airplanes, etc., which have to be removed. The pixels recognized as clouds, must, together with their neighboring pixels, cover an area that is big enough, otherwise, they are removed. The removed pixels could be any kind of disturbances or clouds that are too small to influence the output power of a PV power plant. The output of the cloud recognition is the cloud recognition matrix $C_{r}$. It describes only the clouds big enough to influence the output power of a PV power plant.

### 2.4. Prediction of Cloud Movement in the Vicinity of the Sun

The prediction of cloud movement is required only during the part of the day where the moving clouds could substantially influence the output power of a PV power plant. Thus, a stationary camera with an appropriate wide-angle lens is mounted in such a way as to capture photos of the sky and the Sun during the aforementioned part of the day over the entire year. The coordinates of the Sun’s center are determined in each photo (Subsection 2.2). For the prediction of cloud movement within one minute, the photo is cropped down to a relatively small area around the Sun where the cloud recognition is performed (Subsection 2.3). In order to make the executive code for the prediction of cloud movement, suitable for implementations on low-cost IoT modules, the applied algorithms are minimized regarding the required computational effort. The prediction of cloud movement in the vicinity of the sun is performed in the following steps:

(a) The checkpoints around the Sun are defined;
(b) The cloud covered checkpoint closest to the Sun is identified;
(c) A square area around the cloud covered checkpoint from step (b) is defined;
(d) For the pixels identified as a cloud inside this area, the center of gravity is calculated;
(e) The same procedure as described in step (d) is repeated for the newly taken photo;
(f) The change in the position of the center of gravity between steps (d) and (e) is used to define the cloud’s motion, considering the time between both of the taken photos;
(g) It is checked if the cloud moves towards the Sun’s center and when the Sun’s center will be covered by the cloud;
(h) If the time when the Sun’s center will be covered by the cloud exceeds the preset value, the trigger signal for the reduction of the PV system output power is generated.

**Figure 5:**
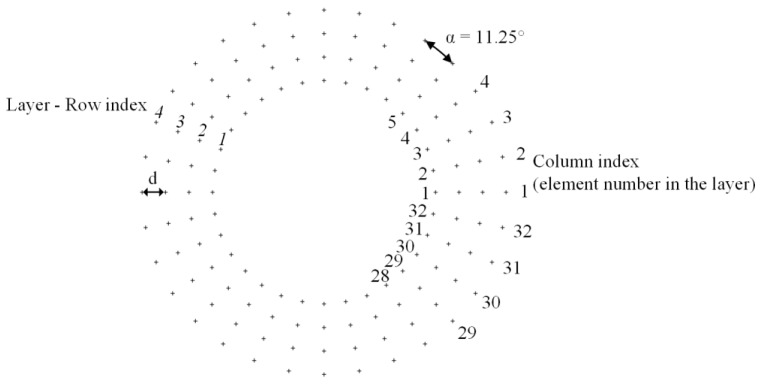
Checkpoints around the Sun and checkpoint matrix indices.

Since the cloud covered checkpoint closest to the Sun is always considered the basis for the prediction of the cloud’s motion, the proposed procedure is able to predict the time when the Sun will be covered by the cloud with a reliability and accuracy also sufficient for complex patterns of moving clouds. The area of observation around the Sun’s center does not exceed a radius 4 to 12 times the size of the radius of the Sun. The pixels representing the cloud closest to the Sun in the cloud recognition matrix are identified first. In order to do this, the checkpoint matrix is defined, containing 128 checkpoints. The selected size of the checkpoint matrix represents a compromise between a low number of checkpoints and the required accuracy, which is sufficient to prevent clouds with complex geometry from covering the Sun without being detected. The checkpoint matrix elements are distributed in four radially equidistant layers, measured from the center of the Sun. Each layer contains 32 angularly equidistant points, as shown in Figure 5. The distance between the layers is marked with *d,* whilst the angle between two neighboring points in the same layer is marked with α = 360°/32 = 11.25°. The checkpoint matrix contains four rows representing layers and 32 columns representing the points in the individual layers. The row and column indices in the checkpoint matrix correspond with the markings shown in Figure 5. The coordinates of the checkpoints are used as a lookup table.

The algorithm for checking the presence of clouds in the vicinity of the Sun starts with internal layer 1 and element 1, which correspond to row 1 and column 1 in the checkpoint matrix. If the elements in the cloud recognition matrix $C_{r}$ at the coordinates, given by the actual row and column in the checkpoint matrix, are set to 1, the presence of a cloud is confirmed at the given checkpoint. If not, the procedure is repeated for the next column (element). If the presence of a cloud cannot be confirmed for any column (element) in the given layer, the layer index is increased and the entire procedure is repeated for all columns (elements) in all layers. When the indices row = 4 and column = 32 are reached and the presence of a cloud is not confirmed, one cycle of the algorithm is completed. After several seconds a new photo is taken and the entire procedure is repeated.

**Figure 6:**
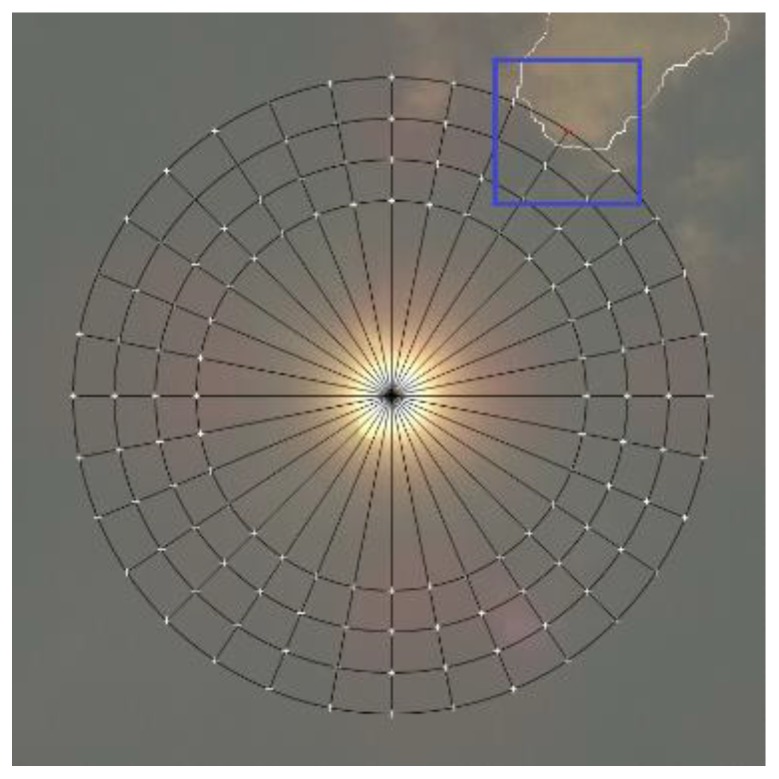
The checkpoint where the presence of a cloud is confirmed and the area around it.

If the presence of a cloud is confirmed, the coordinates of the checkpoint are stored as $(x_{posit}$,$y_{posit})$ and the algorithm is activated for determining the direction of cloud movement. Around the point $(x_{posit}$,$y_{posit})$, where the presence of a cloud is confirmed, a rectangular area with the size of 2m × 2m pixels is defined as shown in Figure 6. This area is confined by the points ($x_{start}$,$\;y_{start}$) and ($x_{end}$, $y_{end}$) the coordinates of which are defined by Equations (5) to (8):

(5) $$x_{start}=x_{posit}-m$$

(6) $$y_{start}=y_{posit}-m$$

(7) $$x_{end}=x_{posit}+m$$

(8) $$x_{end}=y_{posit}+m$$

Inside this area, the center of gravity is calculated only for the surface consisting of the pixels belonging to a cloud which is marked in the cloud recognition matrix. The center of gravity is given by the coordinates ($x_{ave\_p}$,$y_{ave\_p}$) defined by Equations (9) and (10), where $x_{p}$ and $y_{p}$ are the coordinates of the pixels belonging to a cloud inside the area confined by the points ($x_{start}$,$\;y_{start}$) and ($x_{end}$, $y_{end}$), whilst $n_{p}$ is the number of such pixels:

(9) $$x_{ave_{p}}=\frac{\sum_{x_{start}}^{x_{end}}x_{p}}{n_{p}}$$

(10) $$y_{ave\_p}=\frac{\sum_{y_{start}}^{y_{end}}y_{p}}{n_{p}}$$

**Figure 7:**
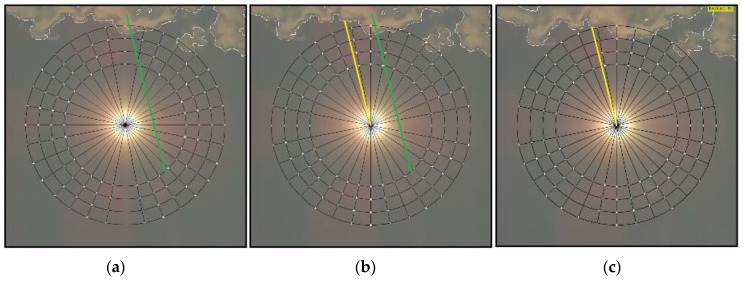
Photos of the sky with the sun, clouds, marked checkpoints and the direction of cloud movement: (**a**) Direction of cloud movement marked with a green line; (**b**) Direction of cloud movement marked with a green line, and a yellow line which has the same angle and starts in the sun’s center—the distance to the nearest cloud is determined along the yellow line; (**c**) The distance to the cloud in the newly taken photo is determined along the same yellow line—the time when the cloud will reach the Sun Equation (18) is predicted based on it (for easier visualization, all lines, points, etc. are shown on the original RGB image.).

After the time $t_{p}$ a new photo of the sky is taken. It is cropped immediately down to the area between the points ($x_{start}$,$\;y_{start}$) and ($x_{end}$,$\;y_{end}$), where the cloud recognition is performed as described in Section 2.3. Using the already described procedure, the center of gravity is determined of the surface belonging to a cloud in the cropped down photo. The center of gravity in the step i is marked with $\left(x_{ave_{p}}\left(i\right),\;y_{ave_{p}}\left(i\right)\right)$, whilst the one in the previous step i−1 is marked with $\left(x_{ave_{p}}\left(i-1\right),\;y_{ave_{p}}\left(i-1\right)\right)$. The difference between both centers of gravity gives the vector ($x_{v}$,$y_{v}$), which describes the cloud’s motion. This vector can be considered an optical flow, limited only to the cloud’s center of gravity inside the square area in Figure 6. Its components $x_{v}$ and $y_{v}$ are determined by Equations (11) and (12). They are used in Equations (13) to (17) to determine the angle of the cloud’s movement γ given in degrees. The straight line, defined by the vector ($x_{v}$,$y_{v}$), starting at the center of gravity $\left(x_{ave_{p}}\left(i-1\right),\;y_{ave_{p}}\left(i-1\right)\right)$, is drawn in the green color in Figure 7a,b:

(11) $$x_{v}=x_{ave_{p}}\left(i-1\right)-x_{ave_{p}}\left(i\right)$$

(12) $$y_{v}=y_{ave_{p}}\left(i-1\right)-y_{ave_{p}}\left(i\right)$$

(13) $$\gamma =\arctan\left(\frac{y_{v}}{x_{v}}\right)\cdot \frac{180}{\pi };\;x_{v}>0$$

(14) $$\gamma =(\arctan\left(\frac{y_{v}}{x_{v}}\right)+\pi )\cdot \frac{180}{\pi };(x_{v}<0)\&\left(y_{v}\geq 0\right)$$

(15) $$\gamma =(\arctan\left(\frac{y_{v}}{x_{v}}\right)+\pi )\cdot \frac{180}{\pi }\;;(x_{v}<0)\&(y_{v}<0)$$

(16) $$\gamma =90\;;\left(x_{v}=0\right)\&(y_{v}>0)$$

A cloud detected in the vicinity of the Sun can move towards the Sun’s center $\left(x_{sun},y_{sun}\right)$, which means that it will shortly cover the Sun, or it can pass by without covering the Sun. To distinguish between the two described cases, the angle γ is used, which defines the direction of the cloud’s movement. The coordinates of individual pixels ($x_{p}$,$y_{p}$), placed along a straight line, starting in the Sun’s center $\left(x_{sun},y_{sun}\right)$, with the angle γ, are calculated by Equation (17) for the variable radius *r*, where ∥ ∥ denotes the round function. It starts with the radius $r_{1}$ that belongs to inner layer 1, shown in Figure 5, and ends with the maximum radius $r_{max}$. The increments of the radius are the size of one pixel:

(17) $$\begin{array}{c} x_{p}=x_{sun}+∥r\cdot \cos\left(\gamma \right)∥\\ y_{p}=y_{sun}+∥r\cdot \sin\left(\gamma \right)∥ \end{array}\left(r\;=r_{1},\;r_{1}+1,\;r_{1}+2,\;\ldots ,r_{max}\;\right)$$

For each pixel location ($x_{p}\left(i\right)$,$\;y_{p}\left(i\right)$) in step i, the pixel’s status is checked in the cloud recognition matrix $C_{r}$, which can be done easily with an “**and**” function. If the pixel is recognized as a cloud, the actual radius r is saved as the distance from the Sun’s center to the cloud. If not, the radius *r* is increased by one increment and the described procedure is repeated until the maximal radius $r_{max}$ is reached or a cloud is detected. After that, in both cases, a new photo of the sky is taken, and the described procedure is repeated. For illustration, the straight line, defined with the pixel coordinates ($x_{p}\left(i\right)$,$\;y_{p}\left(i\right)$) Equation (17) is drawn in the yellow color in Figure 7b,c. 

If the time between two taken photos is $t_{p}$, whilst r(i) and r(i−1) are the distances between the Sun’s center and the cloud in the steps i and i−1, the predicted time in which the cloud will cover the sun $t_{prd}$ can be determined by Equation (18):

(18) $$t_{prd}=\;\frac{t_{p}\;\cdot \;r\left(i-1\right)}{r\left(i-1\right)\;-\;r\left(i\right)}$$

To smooth out any substantial changes in the output power of a PV power plant, caused by the clouds moving in front of the Sun, a proper prediction of the cloud’s movement is required 30 to 60 s before the cloud reaches the sun. If the predicted time $t_{prd}$ (18) is longer than 60 s, the movement of the cloud is slow, and there is enough time to repeat the prediction with better accuracy. In such a case, the obtained result is ignored and the entire procedure starts again from the beginning.

Figure 7a,c are used to illustrate how the movement of a cloud that will cover the Sun is predicted. Figure 7 contains photos of the Sun and the clouds moving in the vicinity of the Sun, where the checkpoints, already presented in Figure 5 and Figure 6, are marked clearly. The straight line drawn in green represents the direction of the cloud’s movement determined by the vector $\left(x_{v},y_{v}\right)$ (Equations (11) and (12)). Its starting point is at the center of gravity $\left(x_{ave_{p}}\left(i-1\right),\;y_{ave_{p}}\left(i-1\right)\right)$. Using the direction angle γ (13) to (16) and the Sun’s center $\left(x_{sun},y_{sun}\right)$ as the starting point, the distance to the nearest cloud moving towards the Sun’s center is determined along the yellow straight line drawn in Figure 7b,c. Only the part of the cloud that is closest to the Sun and is moving towards the Sun’s center must be identified, since it will cover the Sun first. The entire system, consisting of hardware and the procedures proposed in this section, is called the cloud passing forecasting system.

## 3. Results

**Figure 8:**
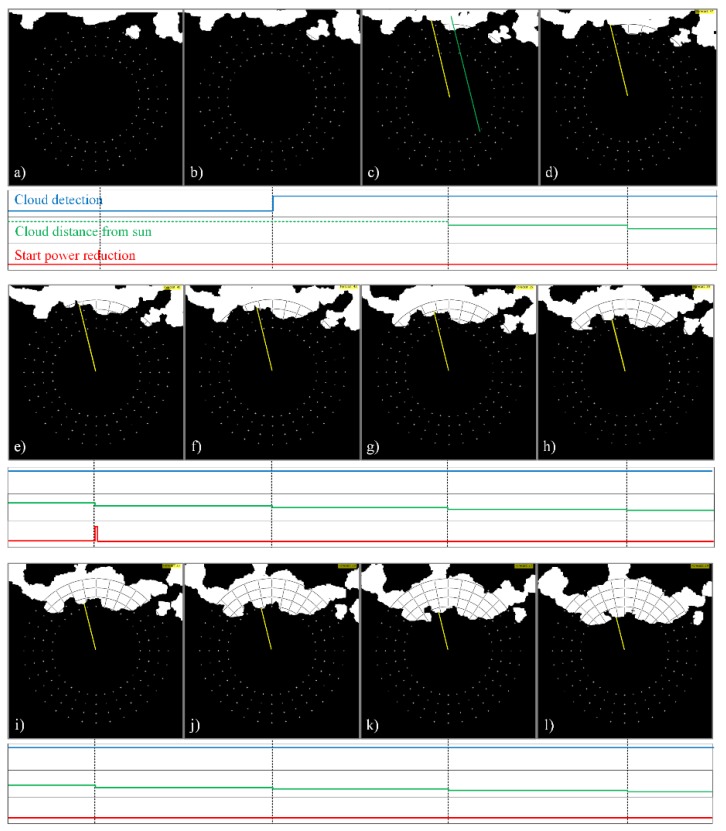
Moving clouds that cover the Sun: a time sequence of processed cloud images (**a**) to (**l**), checkpoints, the signal for the detection of clouds near the sun *“Cloud detection”*, the distance between the Sun and the nearest part of the cloud *“Cloud distance to Sun”* and the signal for the start of output power reduction in a PV power plant *“Start power reduction”*.

**Figure 9:**
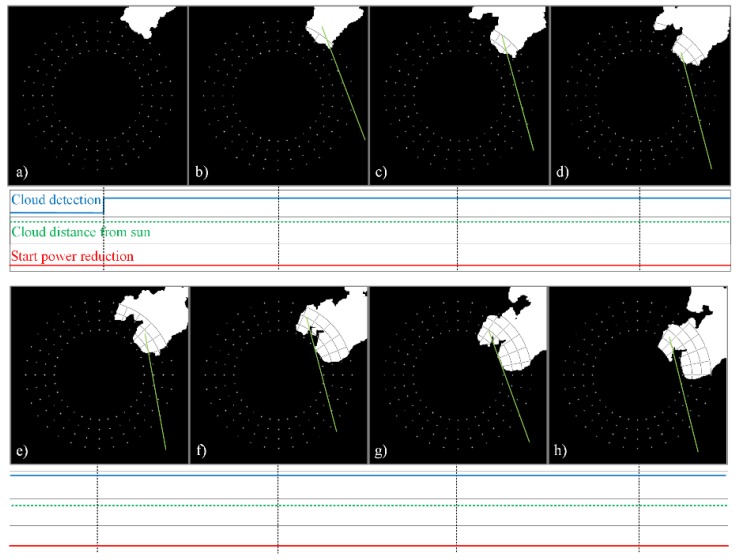
Moving clouds which pass by the Sun without covering it: a time sequence of processed cloud images (**a**) to (**h**), checkpoints, the signal for detection of clouds near the Sun *“Cloud detection”*, the distance between the Sun and the nearest part of the cloud *“Cloud distance to Sun”* and the signal for the start of output power reduction in a PV power plant *“Start power reduction”.*

The sequences of the processed images of the sky, shown in Figure 8 and Figure 9, are used to demonstrate the operation of the proposed cloud passing forecasting system. The time between two taken photos is $t_{p}\;$= 3 s. Figure 8 shows the case when the moving clouds cover the Sun, whilst, in the case shown in Figure 9, the clouds pass by the Sun without covering it.

Figure 8a shows clouds close to the checkpoints, but none of the checkpoints is covered. Therefore, the cloud detection signal is set to the logical value false = 0. In Figure 8b) one of the checkpoints is covered by a cloud. Thus, the cloud detection signal is set to the logical value true = 1 and the area of interest is defined by the points ($x_{start}$,$\;y_{start}$) and ($x_{end}$,$\;y_{end}$) as described in Section 2.4 and shown in Figure 5. The center of gravity of the cloud covered part of the area of interest is determined by Equations (9) and (10).

In the next photo taken the center of gravity is calculated again for the cloud covered part of the same area of interest. Both centers of gravity are used to determine the vector describing the movement of the cloud ($x_{v}$,$\;y_{v}$) (Equations (11) and (12)) and the angle γ (Equations (13)–(16)). They are used to define the green and yellow lines shown in Figure 8c. The green one marks the straight line along which the cloud moves, whilst the yellow straight line is used to measure the distance from the Sun’s center to the part of the cloud moving towards it (see description of Figure 7). Using the same angle γ, the distance to the cloud is measured along the same yellow line shown in Figure 8d–l. It is given in the form of the variable “Cloud distance to Sun”. Based on the distances measured in two images obtained by the image processing of two photos taken in the time difference $t_{p}$, the predicted time $t_{prd}$, in which the cloud will cover the sun, is determined by Equation (18). If this time is shorter than 40 s, a signal for a smooth and slow reduction of the output power is sent to the PV system. 

Figure 9 is used to demonstrate the operation of the proposed cloud passing forecasting system in the case of clouds that pass by the Sun without covering it. Figure 9a shows the cloud that covered a checkpoint which started the procedure for determining the direction of the cloud’s movement. Therefore, the next picture is taken. The image is processed and the vector is determined describing the cloud’s movement. The result is shown in Figure 9b. Since the cloud does not move towards the Sun’s center, the movement of the cloud is observed, as shown in Figure 9c–h. However, no action is taken as long as the cloud is not moving towards the Sun’s center. Some additional results that demonstrate the operation of the proposed system are given in the Appendix A. 

**Figure 10:**
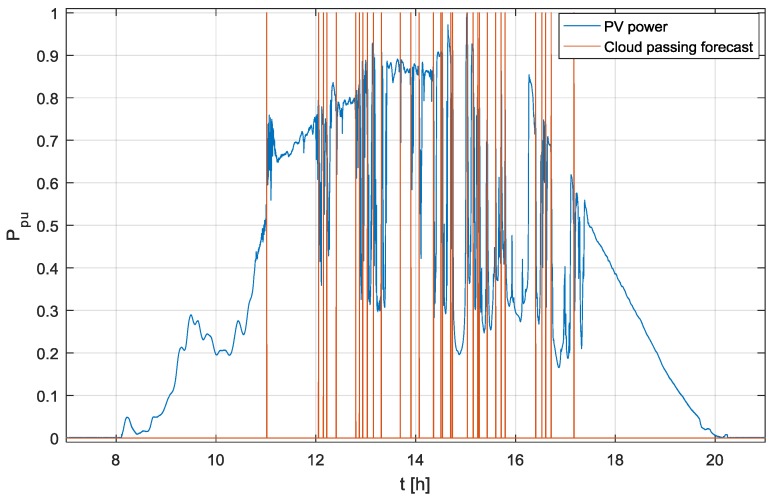
The output power measured on the test PV power plant and the output signal of the proposed cloud passing forecasting system.

**Figure 11:**
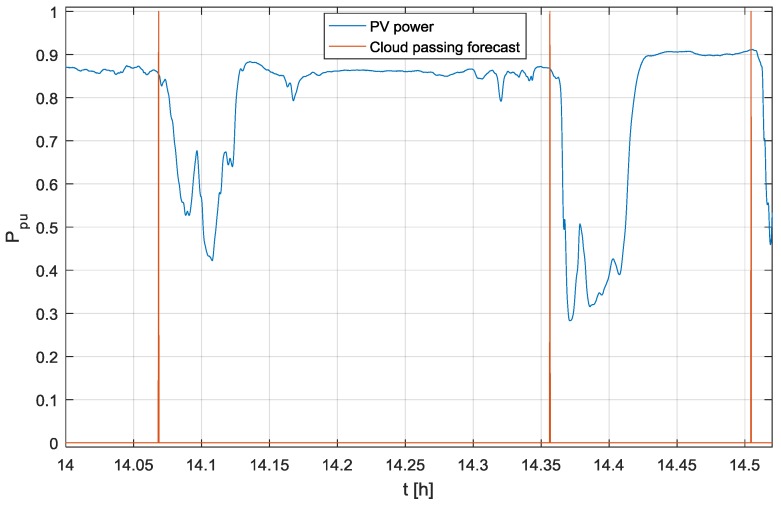
The zoomed-in output power measured on the test PV power plant and the output signal of the proposed cloud passing forecasting system.

The proposed cloud passing forecasting system was the subject of rigorous testing under real operation conditions over a duration of several months, where the output of the proposed system was compared with the output of an operating PV power plant. The analyses of wrong predictions were crucial to develop the final version of the cloud passing forecasting system presented in this paper. The results of the testing over one day are shown in Figure 10 and Figure 11.

Figure 10 shows the output power of a test PV power plant in a per unit (p.u) system and the output of the proposed cloud passing forecasting system, where the logical value true = 1 for the variable *“Cloud passing forecast”* means that a cloud will cover the Sun. As is shown in Figure 10, during the discussed day, the clouds passing in front of the Sun caused a reduction of the output power 32 times. The output signals of the proposed cloud passing forecasting system, which was tested during the discussed day, show effective and reliable cloud passing forecasts. This is even more evident in the zoomed-in results presented in Figure 11. 

According to the applied settings, the cloud passing forecasting system should generate the output signal *“Start power reduction”* shown in Figure 8 40 s before the cloud covers the Sun. This time is sufficient to reduce the output power of the PV system smoothly and slowly, before a cloud moving in front of the sun causes its fast change. 

The results shown in Figure 10 are evaluated numerically by the Root Mean Square Error (RMSE, (Equation (19)) and the Mean Biased Error (MBE, Equation (20)):

(19) $$RMSE=\;\sqrt{\frac{1}{m}\overset{m}{\underset{n=1}{\sum }}{\left(t_{M}\left(n\right)-t_{f}\right)}^{2}}$$

(20) $$MBE=\;\frac{1}{m}\overset{m}{\underset{n=1}{\sum }}\left(t_{M}\left(n\right)-t_{f}\right)$$

where  m = 32 is the number of events in which the passing clouds caused the reduction of output power (in Figure 10), n is the event index, $\;t_{f\;}$ = 30 s is the time predicted by the proposed cloud passing forecasting system in which the PV system output power should be reduced by 5% due to the moving clouds, whilst $t_{M}\left(n\right)$ is the measured time in which the PV system output power is reduced by 5%. The calculated values are RMSE = 2.90 s and MBE = 0.433 s. This means the relative values RMSE and MBE are 9.7% and 1.44% respectively. For the given field of applications even the results with RMSE values over 30% are acceptable. 

## 4. Conclusions

This paper proposes a cloud passing forecasting system that can be implemented on low-cost IoT hardware and is suitable for predicting the movement of clouds in the vicinity of the Sun in a time interval of under one minute. This is achieved by the minimization of the computational effort required for image processing. The novelties proposed in this paper are the use of the green and the blue colors in the sun and cloud recognition and the procedures for:

- Detecting clouds in the vicinity of the Sun;
- Determining the cloud’s movement;
- Determining the distance between the cloud and the Sun;
- Predicting the time during which the cloud will cover the Sun.

The results presented have shown that the proposed cloud passing forecasting system can provide a reliable and sufficiently accurate prediction of cloud movement in the vicinity of the Sun. Its output signal can be used to reduce the output power of PV systems smoothly and slowly. The proposed solution is an efficient tool for the prevention of fast changes in the output power of PV systems, whilst its cost is lower than the cost of a single 270 Wp PV module.

## Figures and Tables

**Table 1:** Costs of hardware components.

Item	Price
Raspberry Pi 3 Model B (RPI 3 B)	34 €
Waveshare RPi Camera (G)	29 €
ND 4 Filter 52 mm	3 €
CPL Filter 52 mm	4 €
Total	70 €

**Figure A1:**
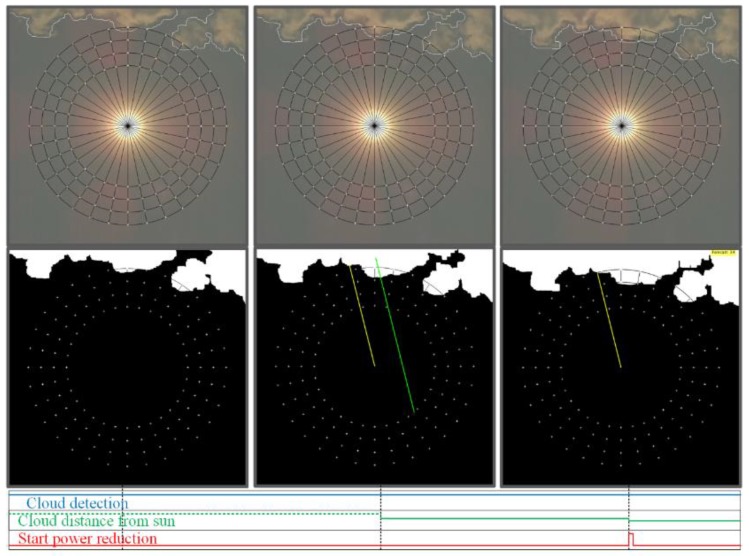
Clouds moving in the vicinity of the Sun and the signals “Cloud detection”, “Cloud distance to Sun” and “Start power reduction” in the cloud passing prediction system.

**Figure A2:**
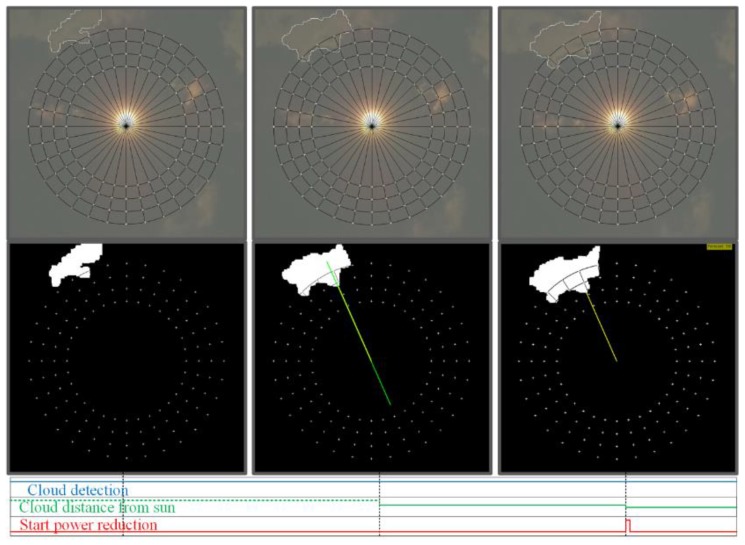
Clouds moving in the vicinity of the Sun and the signals “Cloud detection”, “Cloud distance to Sun” and “Start power reduction” in the cloud passing prediction system.

**Figure A3:**
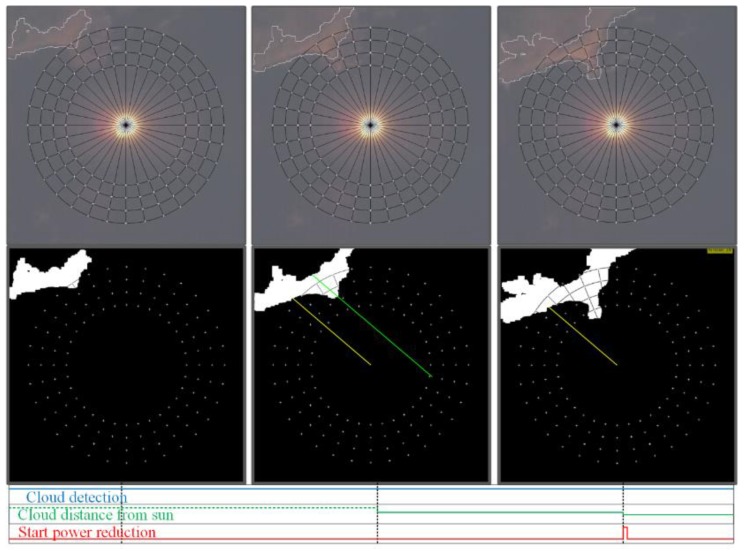
Clouds moving in the vicinity of the Sun and the signals “Cloud detection”, “Cloud distance to Sun” and “Start power reduction” in the cloud passing prediction system.

**Figure A4:**
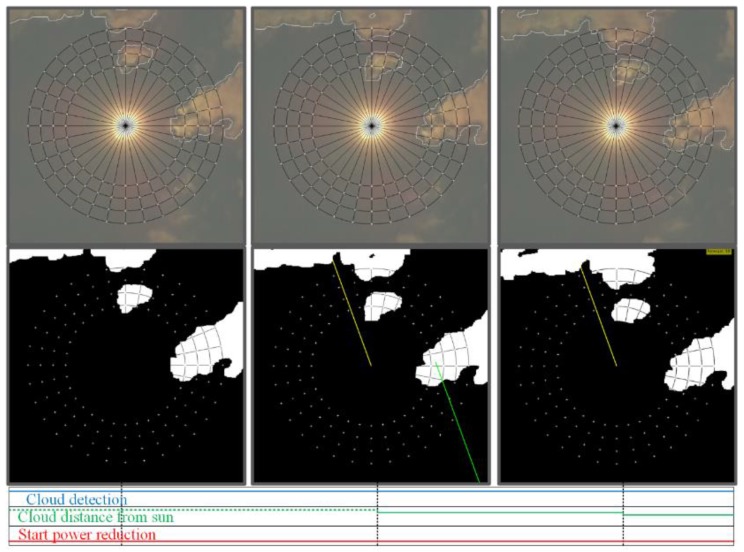
Clouds moving in the vicinity of the Sun and the signals “Cloud detection”, “Cloud distance to Sun” and “Start power reduction” in the cloud passing prediction system.

**Figure A5:**
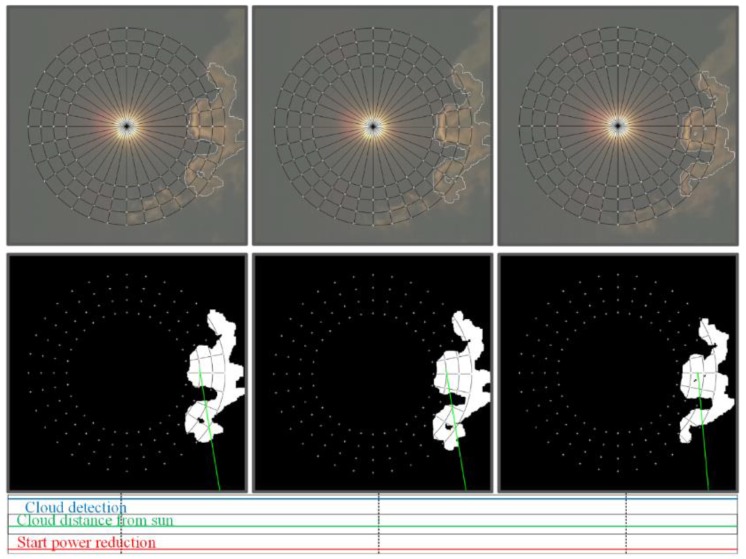
Clouds moving in the vicinity of the Sun and the signals “Cloud detection”, “Cloud distance to Sun” and “Start power reduction” in the cloud passing prediction system.

**Figure A6:**
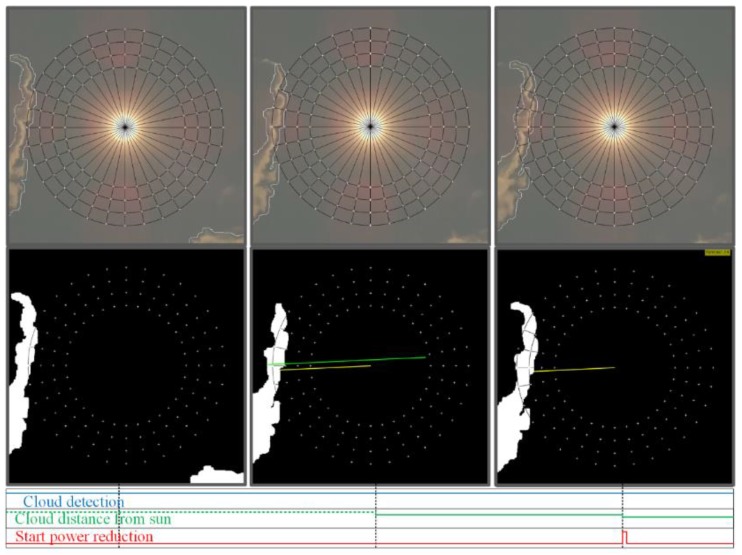
Clouds moving in the vicinity of the Sun and the signals “Cloud detection”, “Cloud distance to Sun” and “Start power reduction” in the cloud passing prediction system.

**Figure A7:**
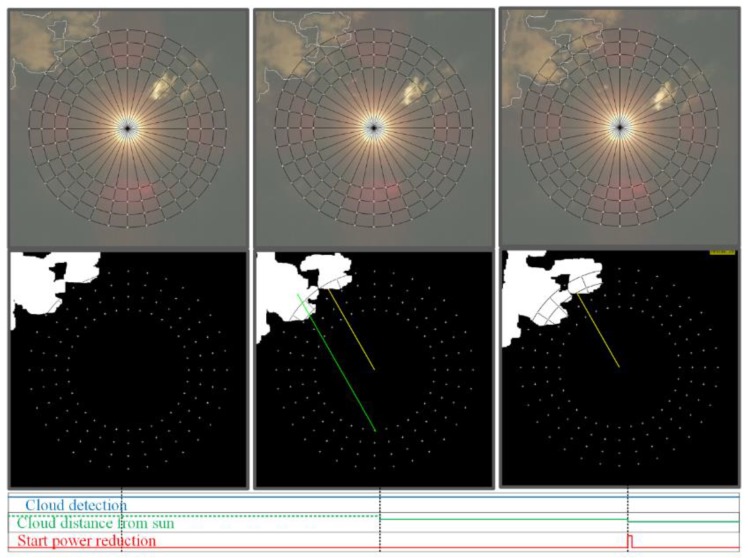
Clouds moving in the vicinity of the Sun and the signals “Cloud detection”, “Cloud distance to Sun” and “Start power reduction” in the cloud passing prediction system.

**Figure A8:**
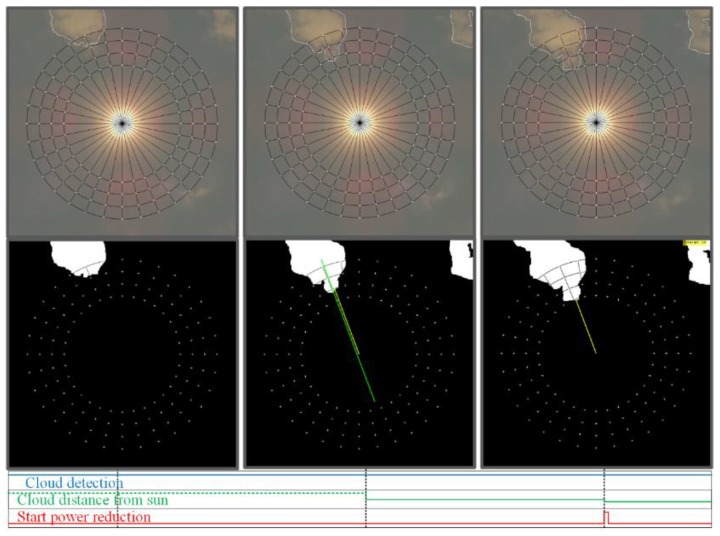
Clouds moving in the vicinity of the Sun and the signals “Cloud detection”, “Cloud distance to Sun” and “Start power reduction” in the cloud passing prediction system.

## Appendix A

Although the most relevant results of the testing performed on the proposed cloud passing forecasting system under real operating conditions are shown in Figure 10 and Figure 11 for one day, the results presented in Figure A1, Figure A2, Figure A3, Figure A4, Figure A5, Figure A6, Figure A7 and Figure A8, for a series of different real life patterns of moving clouds, should help to demonstrate the operation of the proposed system. The meaning of the signals *“Cloud detection”*, *“Cloud distance to Sun”* and *“Start power reduction”* is the same as in Figure 8 and Figure 9.
